# Clinical analysis of chemo-resistance risk factors in endometriosis associated ovarian cancer

**DOI:** 10.1186/s13048-018-0418-8

**Published:** 2018-05-29

**Authors:** Tong Ren, Ting-Ting Sun, Shu Wang, Jian Sun, Yang Xiang, Keng Shen, Jing-He Lang

**Affiliations:** 1 Department of Obstetrics and Gynecology, Peking Union Medical College Hospital, Chinese Academy of Medical Science and Peking Union Medical College, 1 ShuaiFuYuan, DongCheng District, Beijing, 100730 China; 2 Department of Pathology, Peking Union Medical College Hospital, Chinese Academy of Medical Science and Peking Union Medical College, Beijing, People’s Republic of China

**Keywords:** Chemo-resistance, Risk factors, Endometriosis associated ovarian cancer

## Abstract

**Background:**

To analyze the clinical characteristics and chemo-resistance related factors of patients with resistant and non-resistant endometriosis-associated ovarian cancer (ovarian clear cell carcinoma and endometrioid carcinoma) by reviewing the data of epithelial ovarian cancer patients who received initial treatment in our hospital over a 12-year period.

**Results:**

Among the 304 patients, 17.1% were seen with platinum-based drug resistance. The ROC curve of continuous variables was drawn according to resistance situation, then they were grouped by age (< 48 or ≥ 48 years), tumor size (< 7 cm or ≥ 7 cm) and Ca125 (< 90 and ≥ 90 U/ml). In univariate analysis, age ≥ 48 years, initial symptom of abdominal distension or weight loss, abnormal preoperative serum Ca125, Ca125 < 90 U/ml, advanced FIGO stage, absence of endometriosis, bilateral tumors, lack of lymphadenectomy, positive lymph nodes, unsatisfactory initial cytoreduction surgery and history of breast cancer were all related to drug resistance in ovarian cancer. In multivariate analysis, advanced stage, lack of lymphadenectomy, positive lymph nodes and history of breast cancer were independent risk factors related to platinum-based drug resistance (*P* < 0.05).

**Conclusion:**

For patients of endometriosis-related ovarian cancer, platinum-based drug resistance were associated with advanced FIGO stage, lack of lymphadenectomy, positive lymph nodes and history of breast cancer.

## Background

At present, many scholars regard endometriosis-associated ovarian cancer(EAOC) as a special pathological type of epithelial ovarian cancer(EOC), including ovarian clear cell carcinoma (OCCC) and endometrioid carcinoma. Studies have reported that the clinical features and prognosis of patients with EAOC were different from those with other EOCs. Compared with ovarian serous carcinoma, the onset age of OCCC was younger (55 vs 64 years; median age) [1]. A study comparing early ovarian cancer and advanced ovarian cancer (I/II vs. III/IV) reported that 57–81% of OCCC were diagnosed in early stage (I/II) [2]. Since most patients of OCCC were with unilateral pelvic mass of early stage, their prognosis was better than other types of EOC. The 5-year survival rate of OCCC patients with early stage was higher than 80% [3]. However, the prognosis of those with advanced stage was poorer than that of other EOCs with the 5-year survival rate about 20%, which might be related to the resistance to platinum-based chemotherapy [4]. The clinical risk factors of chemo-resistance in patients with EAOC have not been reported. By retrospectively analyzing the data of EOC patients who received initial treatment in our hospital over a 12-year period, this study intended to analyze the clinical features and chemo-resistance related factors of patients with resistant and non-resistant EAOC (OCCC and endometrioid carcinoma), expecting to provide references for the guidance of clinical treatments and the prediction of survival outcomes.

## Results

**Fig. 1:**
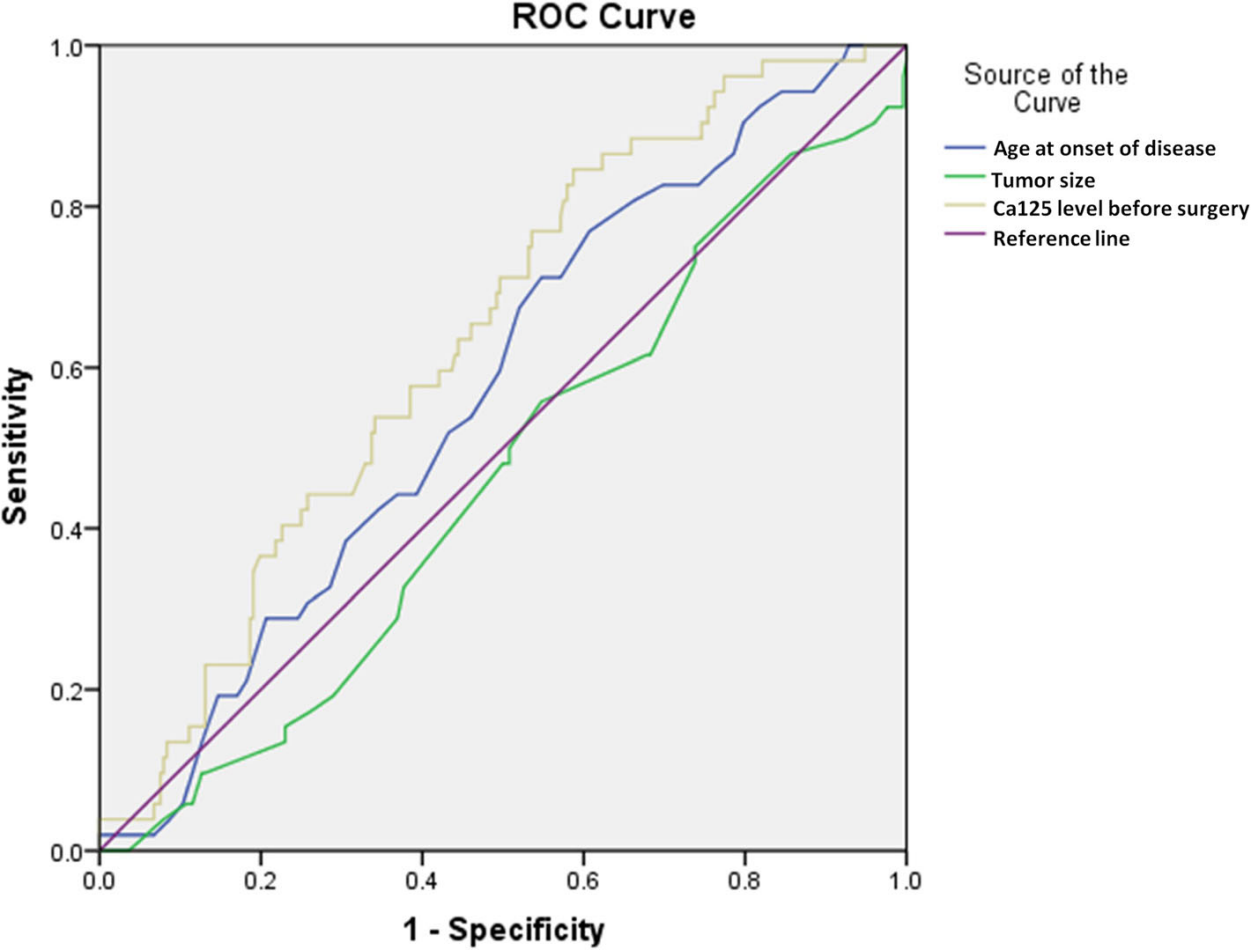
ROC curve for age of disease onset, tumor size, pre-surgery Ca125 in the occurrence of chemo-resistance

A total of 304 patients diagnosed with EAOC and treated in PUMCH were identified, 52(17.1%) of which were seen with platinum-based drug resistance and the rest 252(82.9%) were in the non-resistant group. Table 1 showed the demographic and clinical characteristics of all the patients. In this study, based on the ROC curve of chemo-resistance, the above continuous variables were grouped by age, tumor size and Ca125 level[age: < 48 or ≥ 48 years; tumor size: < 7 cm or ≥ 7 cm; Ca125: < 90 U/L or ≥ 90 U/L], as seen in Fig. 1. Univariate analysis showed that compared with the non-resistant group, there were more patients with age ≥ 48 years in the chemo-resistant group (71.15% vs 54.76%, *P* = 0.0294), more with symptoms of abdominal distension (32.69% vs 17.46%, *P* = 0.0125) and emaciation (11.54% vs 1.59%, *P* = 0.0002), fewer with normal level of Ca125 (3.85% vs 21.03%, *P* = 0.0034) but more Ca125 < 90 U/L (84.62% vs 58.73%, *P* = 0.0004), more advanced FIGO stage(III + IV) (80.77% vs 30.56%, *P* < 0.001), more high-grade tumor(62.07% vs 40.76%; *P* = 0.0128), fewer coexisting endometriosis (9.62% vs 25.4%, *P* = 0.0134), more bilateral tumors (44.23% vs 27.38%, *P* = 0.0160), more positive LNs (30.77% vs 9.52%, *P* < 0.001), fewer lymphadenectomy (34.62% vs 13.1%, *P* = 0.0002), more residual tumor > 1 cm and more history of breast cancer (7.69% vs 1.59%, *P* = 0.0123). Meanwhile, there was no statistically significant difference seen in menopause status, number of pregnancies and labors, tumor size, histologic type, coexisting endometrial lesions, history of hysterectomy or tubal sterilization and history of diabetes mellitus or hypertension, as showed in Table 1 (*P* > 0.05).

**Table 1:** Clinical and morphological characteristics of patients with or without chemo-resistance

Variable	Category	Number(%)	Chemo-sensitive(%)	Chemoresistance(%)	*P*
		304	252(82.9%)	52(17.1%)	
Age	< 48	129(42.43)	114(45.24)	15(28.85)	0.0294*
	≥48	175(57.57)	138(54.76)	37(71.15)	
Menopausal status	Pre	161(52.96)	139(55.16)	22(42.31)	0.0909
	Post	143(47.04)	113(44.84)	30(57.69)	
Gravidity	< 1	35(11.51)	31(12.3)	4(7.69)	0.3431
	> = 1	269(88.49)	221(87.7)	48(92.31)	
Parity	< 1	51(16.78)	46(18.25)	5(9.62)	0.1290
	> = 1	253(83.22)	206(81.75)	47(90.38)	
Abdominal pain	No	210(69.08)	174(69.05)	36(69.23)	0.9016
	Yes	93(30.59)	77(30.56)	16(30.77)	
Bloating	No	243(79.93)	208(82.54)	35(67.31)	0.0125*
	Yes	61(20.07)	44(17.46)	17(32.69)	
Palpable mass	No	223(73.36)	182(72.22)	41(78.85)	0.3253
	Yes	81(26.64)	70(27.78)	11(21.15)	
Incidental finding	No	256(84.21)	208(82.54)	48(92.31)	0.0786
	Yes	48(15.79)	44(17.46)	4(7.69)	
Irregular menstruation	No	275(90.46)	226(89.68)	49(94.23)	0.3094
	Yes	29(9.54)	26(10.32)	3(5.77)	
Postmenopausal bleeding	No	285(93.75)	235(93.25)	50(96.15)	0.4316
	Yes	19(6.25)	17(6.75)	2(3.85)	
Emaciation	No	294(96.71)	248(98.41)	46(88.46)	0.0002*
	Yes	10(3.29)	4(1.59)	6(11.54)	
Abnormal vaginal discharge	No	302(99.34)	250(99.21)	52(100)	0.5192
	Yes	2(0.66)	2(0.79)	0(0)	
Ca125 in normal range(< 35 U/L)	No	249(81.91)	199(78.97)	50(96.15)	0.0034*
	Yes	55(18.09)	53(21.03)	2(3.85)	
CA125group	< 90	112(36.84)	104(41.27)	8(15.38)	0.0004*
	≥90	192(63.16)	148(58.73)	44(84.62)	
Early or late Stage	FIGO I + II	185(60.86)	175(69.44)	10(19.23)	<.0001*
	FIGO III + IV	119(39.14)	77(30.56)	42(80.77)	
FIGO stage^a^	I	141(46.38)	136(53.97)	5(9.62)	<.0001*
	II	44(14.47)	39(15.48)	5(9.62)	
	III	104(34.21)	69(27.38)	35(67.31)	
	IV	15(4.93)	8(3.17)	7(13.46)	
Tumor size group	< 7	80(26.32)	66(26.19)	14(26.92)	0.9130
	≥7	224(73.68)	186(73.81)	38(73.08)	
Pathology Grade	G1	45(24.19)	44(28.03)	1(3.45)	0.0128*
	G2	59(31.72)	49(31.21)	10(34.48)	
	G3	82(44.09)	64(40.76)	18(62.07)	
Pathology type	Endometrioid	186(61.18)	157(62.30)	29(55.77)	0.435
	Clear cell	118(38.82)	95(37.70)	23(44.23)	
Endometriosis	No	235(77.30)	188(74.6)	47(90.38)	0.0134*
	Yes	69(22.70)	64(25.4)	5(9.62)	
Side of tumor	Unilateral	212(69.74)	183(72.62)	29(55.77)	0.0160*
	Bilateral	92(30.26)	69(27.38)	23(44.23)	
LN metastasis	No	213(70.07)	195(77.38)	18(34.62)	<.0001*
	Yes	40(13.16)	24(9.52)	16(30.77)	
	unclear	51(16.78)	33(13.1)	18(34.62)	
LN dissection	No	51(16.78)	33(13.1)	18(34.62)	0.0002*
	Yes	253(83.22)	219(86.9)	34(65.38)	
Residual disease	No	231(75.99)	207(82.14)	24(46.15)	<.0001*
	Yes	73(24.01)	45(17.86)	28(53.85)	
TH	No	297(97.7)	246(97.62)	51(98.08)	0.8411
	Yes	7(2.3)	6(2.38)	1(1.92)	
Tubal ligation	No	290(95.39)	243(96.43)	47(90.38)	0.0583
	Yes	14(4.61)	9(3.57)	5(9.62)	
Sterilization surgery	No	283(93.09)	237(94.05)	46(88.46)	0.1481
	Yes	21(6.91)	15(5.95)	6(11.54)	
Endometrial disorder	No	252(82.89)	208(82.54)	44(84.62)	0.7174
	Yes	52(17.11)	44(17.46)	8(15.38)	
Variable endometrial disorder	No	252(82.89)	207(82.14)	45(86.54)	0.7998
	EP	27(8.88)	23(9.13)	4(7.69)	
	EIN	22(7.24)	19(7.54)	3(5.77)	
	EC	3(0.99)	3(1.19)	0(0)	
Breast cancer	No	296(97.37)	248(98.41)	48(92.31)	0.0123*
	Yes	8(2.63)	4(1.59)	4(7.69)	
HT	No	250(82.24)	208(82.54)	42(80.77)	0.7610
	Yes	54(17.76)	44(17.46)	10(19.23)	
DM	No	286(94.08)	236(93.65)	50(96.15)	0.4862
	Yes	18(5.92)	16(6.35)	2(3.85)	

^a^According to the classification system of FIGO staging (2013 version)

*Abbreviation*: *Ca125* cancer antigen 125, *EM* endometriosis, *LN* lymph node, *TH* total hysterectomy, *EP* endometrial polyps, *EIN* endometrial intraepithelial neoplasm, *EC* endometrial cancer, *HT* hypertension, *DM* diabetic mellitus

*P < 0.05

In multivariate analysis however, only FIGO stage (*P* = 0.004), positive LNs (*P* = 0.040), lymph nodes resection (*P* = 0.016) and history of breast cancer (*P* = 0.044) were independent risk factors of platinum-based drug resistance, while onset age, initial symptoms, Ca125 level, coexisting endometriosis and residual tumors were not, as seen in Table 2.

**Table 2:** Multivariate analysis of risk factors of chemo-resistance among endometriosis related ovarian cancer patients

	B	S.E.	P	OR(95%CI)
Age	.337	.395	.393	1.4(0.65~ 3.04)
Bloating	−.057	.401	.886	0.94(0.43~ 2.07)
Emaciation	1.447	.791	.067	4.25(0.9~ 20.02)
Ca125 in normal range	.728	.797	.361	2.07(0.43~ 9.87)
EM	−.016	.573	.978	0.98(0.32~ 3.03)
FIGO stage^a^	1.422	.497	.004*	4.15(1.57~ 10.97)
LN metastasis	.980	.477	.040*	2.66(1.05~ 6.78)
LN dissection	1.059	.440	.016*	2.88(1.22~ 6.82)
Residual disease	.396	.405	.328*	1.49(0.67~ 3.29)
Breast cancer	1.851	.918	.044*	6.37(1.05~ 38.49)
Constant	−5.388	1.018	.000	

*The difference reached statistical significance. *P* values were cultivated by Cox regression analysis. The overall test of the above model showed the model was significance, *p* < 0.0001

^a^According to the classification system of FIGO staging (2013 version)

*Abbreviation*: *Ca125* cancer antigen 125, *EM* endometriosis, *LN* lymph node

## Discussion

This study regarded OCCC and ovarian endometrioid carcinoma as a whole of EAOC for the first time and investigated the clinicopathological risk factors of platinum-based chemoresistance. Univariate analysis showed that age, higher level of Ca125, advanced FIGO stage, high-grade tumor, absence of endometriosis, bilateral tumors, lack of lymphadenectomy, positive LNs, residual lesion > 1 cm and history of breast cancer were related to chemoresistance. However, multivariate analysis showed that FIGO stage, lack of lymphadenectomy, positive LNs and history of breast cancer were independent risk factors associated with drug resistance to platinum in patients with such type of EOC.

A large number of previous studies focused on the drug resistance in OCCC. Some retrospective studies have shown that OCCC was resistant to traditional platinum-based chemotherapy regimens with an objective effective rate of 11–27%, while the response rate of serous adenocarcinoma (SAC) was 73–81%, significantly higher than that of OCCC [5–7]. Utsunomiya et al. found that the effective rate of paclitaxel plus carboplatin (TC) regimen in patients with OCCC was not high either [8]. Rauh-Hain et al. reported that the response rate of 121 OCCC patients treated with first-line platinum-based chemotherapy regimens was 79 and 24% of the patients relapsed within 6 months after the last cycle of chemotherapy of initial treatments [9]. Moreover, their results showed that unsatisfactory cytoreductive surgery and wide dissemination of tumors were significantly associated with platinum resistance by multivariate logistic regression analysis. On the other hand, Liang et al. have reported that advanced stage, poor differentiation, LN positivity, CA125 level > 1000 U/mL and suboptimal cytoreductive surgery would lead to drug resistance or partial sensitivity to chemotherapy during the treatment of OCCC. These results were not in full accord with the findings of this study [10].

The mechanism of drug resistance to chemotherapy in OCCC was complex, which might be related to the low proliferation rate of the tumors, the increase of damage to DNA repair activity, the up-regulation of growth factor signaling pathway and the abnormal expression of microtubule-disaggregated protein, etc. Studies have shown that the high resistance of OCCC to chemotherapy might be related to its low cell proliferation rate [4]. Itamochi et al. reported that the doubling time for tumor cells of OCCC was significantly longer than that of SAC (61.4 vs 29.8 h) [11]. Ki-67 protein was expressed at various stages of the cell cycle, representing the proliferative activity of the cells, and its expression in OCCC was significantly lower than that in SAC. In addition, the Ki-67 labelling index (LI) in patients that are resistant to platinum-based chemotherapy was significantly lower than it in those sensitive (15.3% vs 30.2%) [4]. As known, platinum-based drugs inhibited the proliferation of tumor cells mainly by hindering the replication of DNA. Therefore, the low proliferation rate of OCCC cells enabled them to some extent to be tolerant to platinum-based drugs targeting on DNA, which suggested that the chemoresistance of OCCC might be associated with its low proliferation rate [12].

Previous studies have showed tumors lack of DNA mismatch repair (MMR) system were highly resistant to certain methylated drugs of chemotherapy in vitro [13]. The function of MMR is to correct mistakes in DNA replication and play an important role in the sensitivity of DNA damage factors. MMR deficiency might result from germline mutations of two major MMR genes, such as hMLH1 and hMSH2, as well as epigenetic silencing due to the methylation of the hMLH1 promoter, leading to inactivation of the gene system. Cai et al. reported that the high expression of mutations in hMLH1 and hMSH2 existed in OCCC and was associated with its development [14]. In addition, Niimi K et al. also reported that the expression of DNA damage repair related protein REV7 in OCCC was significantly higher than that in other types of EOCs (73.5% vs 53.4%), and the knockdown of REV gene could induce apoptosis and DNA damage in tumor cells, leading to a significant improvement of chemoresistance to cisplatin in OCCC [15]. On the other hand, Itamochi H et al. reported that the use of CHK inhibitors could improve the drug resistance of OCCC to cisplatin [16], while the main function of cell cycle checkpoint kinase (CHK) was to regulate the synthesis of DNA in tumor cells.

In addition, the up-regulation of growth factor signaling pathway is related to drug resistance in tumor. As cell surface receptor tyrosine kinase, epidermal growth-factor receptor (EGFR) can activate the mitogen-activated protein kinase pathway, thus inhibiting the apoptosis induced by chemotherapy drugs [17]. An immunohistochemical study showed that EGFR could be found in 61% of CCC, and the overexpression of EGFR might be related to chemotherapy resistance and poor prognosis of ovarian cancer [18]. Siddiqui GK et al. reported that in platinum-resistant group, the proportion of patients with high expression of VEGF in tumor tissues was significantly higher than it in the platinum-sensitive group (86% vs 2%), suggesting that the resistance of EOC to platinum-based chemotherapy was related to VEGR expression [19]. Mabuchi S et al. confirmed in vitro that the expression of VEGF in cisplatin-resistant OCCC cell lines was significantly higher than that in cisplatin-sensitive OCCC cell lines, suggesting that the generation of drug resistance might be related to the angiogenesis in the tumors [20]. According to previous literatures, the expression of HER2 in OCCC was much higher than it in other major histological types of EOCs, and tumors with overexpression of HER2 showed low sensitivity to traditional anti-tumor drugs, leading to the poor prognosis of these patients [21, 22].

Studies about the drug resistance in ovarian endometrioid carcinoma was limited. Pylväs-Eerola et al. have reported that the preoperative level of 8-hydroxy-2-deoxyguanosine in patients were significantly associated with chemoresistance of ovarian endometrioid carcinoma, which could be used as a factor for resistance prediction [23].

Our results showed that histologic type indicating OCCC or endometrioid carcinoma was not an independent risk factor of platinum-based drug resistance (55.77% vs 44.23%, *P* = 0.435), which was not consistent with previous studies, suggesting that the platinum-based drug resistance in these two types of tumors might have similarities. More molecular biological researches were expected to explore the molecular mechanism of chemoresistance in EAOC.

## Conclusion

This study regarded endometriosis-related OCCC and endometrioid carcinoma as a whole of EAOC for the first time and analyzed the clinicopathological risk factors of platinum-based chemoresistance. Multivariate analysis showed that FIGO stage, positive LNs, lack of lymphadenectomy and history of breast cancer were independent risk factors of drug resistance in patients with EAOC. This finding is of certain value for predicting platinum-based drug resistance in the treatment of EAOC, which may give some instructions for designing individualized chemotherapy regimens. In the future, it is expected to carry out more accurate molecular typing for such patients through molecular biological study, which can be used to guide precise medication of chemotherapy drugs for patients with ovarian cancers.

## Methods

This is a retrospective study conducted at Department of Obsterics and Gynecology, Peking Union Medical College Hospital(PUMCH). We identified all the 304 patients who received surgical treatments and postoperative chemotherapy from January 2000 to December 2012, including 118 cases of OCCCs and 186 cases of endometrioid carcinomas confirmed by postoperative histopathology. All patients with EAOC of early stage (stage I and II) have received completed staging surgery; and those of advanced stage (stage III and IV) have undergone optimal CRS, except for those with unresectable tumors who received suboptimal CRS. They all received adjuvant chemotherapy of platinum-based regimens after primary surgery.

The clinicopathologic data of patients with EAOC were collected, including age, initial symptoms, menopause status, number of pregnancies and labors, previous medical complications, preoperative CA125 level, FIGO stage, tumor size, laterality, histologic type, grade, coexisting endometriosis, lymph nodes(LNs) metastasis, lymphadenectomy (the resection of pelvic LNs with or without para-aortic LNs), residual tumor, history of hysterectomy and tubal ligation, coexisting endometrial lesions (including endometrial polyps, atypical hyperplasia and endometrial cancer), history of breast cancer, history of hypertension and diabetes. Platinum-based drug resistance in ovarian cancer was defined as a definite progression or recurrence of disease within 6 months after completing the last cycle of chemotherapy for initial treatments. In this study, we defined ovarian cancer concurrent with endometriosis as the presence of ovarian cancer and endometriosis identified histologically in the same ovary, the presence of endometriosis in one ovary and ovarian cancer in the contralateral ovary, or the presence of ovarian cancer and extraovarian pelvic endometriosis (eg, peritoneal endometriosis).

SPSS 22.0 was used for statistical analysis. Continuous variables were analyzed using an independent-sample *t* test. Categorical variables were analyzed using χ^2^ test or Fisher’s exact test. Odds ratio (OR) and 95% confidence interval (CI) were calculated. The effects of clinicopathological characteristics on chemoresistance in EAOC were assessed using logistic regression models through univariate and multivariate analysis. Receiver Operating Characteristic (ROC) curve was constructed to define the optimal cutoff value for stratifying and grouping. All statistical tests were two-sided and differences were considered statistically significant at *P* < 0.05.

## Abbreviations

Ca125: Cancer antigen 125
DFS: Disease-free survival
EAOC: Endometriosis-associated ovarian cancer
EAOCCC: Endometriosis-associated ovarian clear cell carcinoma
EAOEC: Endometriosis-associated ovarian endometrioid carcinoma
OS: Overall survival
